# HDAC3 Mediates the Inflammatory Response and LPS Tolerance in Human Monocytes and Macrophages

**DOI:** 10.3389/fimmu.2020.550769

**Published:** 2020-10-05

**Authors:** Mohammed Ghiboub, Jing Zhao, Andrew Y. F. Li Yim, Ronald Schilderink, Caroline Verseijden, Patricia H. P. van Hamersveld, Jose M. Duarte, Theodorus B. M. Hakvoort, Iris Admiraal, Nicola R. Harker, David F. Tough, Peter Henneman, Menno P. J. de Winther, Wouter J. de Jonge

**Affiliations:** ^1^Tytgat Institute for Liver and Intestinal Research, Amsterdam Gastroenterology & Metabolism, Amsterdam University Medical Centers, University of Amsterdam, Amsterdam, Netherlands; ^2^Epigenetics Discovery Performance Unit, Immunoinflammation Therapy Area Unit, Medicines Research Centre, GlaxoSmithKline, Stevenage, United Kingdom; ^3^Genome Diagnostics Laboratory, Amsterdam Reproduction & Development, Department of Clinical Genetics, Amsterdam University Medical Centers, University of Amsterdam, Amsterdam, Netherlands; ^4^Adaptive Immunity Research Unit, Medicines Research Centre, GlaxoSmithKline, Stevenage, United Kingdom; ^5^Department of Medical Biochemistry, Amsterdam University Medical Centers, University of Amsterdam, Amsterdam, Netherlands; ^6^Department of Medicine, Institute for Cardiovascular Prevention (IPEK), Munich, Germany; ^7^Department of Surgery, University of Bonn, Bonn, Germany

**Keywords:** IFNγ, macrophages, HDAC3 inhibition, LPS tolerance, inflammatory response

## Abstract

Histone deacetylases (HDACs) are a group of enzymes that control histone deacetylation and bear potential to direct expression of large gene sets. We determined the effect of HDAC inhibitors (HDACi) on human monocytes and macrophages, with respect to their polarization, activation, and their capabilities of inducing endotoxin tolerance. To address the role for HDACs in macrophage polarization, we treated monocytes with HDAC3i, HDAC6i or pan-HDACi prior to polarization into M1 or M2 macrophages using IFNγ or IL-4 respectively. To study the HDAC inhibition effect on cytokine expression, macrophages were treated with HDACi prior to LPS-stimulation. TNFα, IL-6, and p40 were measured with ELISA, whereas modifications of Histone 3 and STAT1 were assessed using western blot. To address the role for HDAC3 in repeated LPS challenge induction, HDAC3i or *HDAC3* siRNA was added to monocytes prior to incubation with IFNγ, which were then repeatedly challenged with LPS and analyzed by means of protein analyses and transcriptional profiling. Pan-HDACi and HDAC3i reduced cytokine secretion in monocytes and M1 macrophages, whereas HDAC6i yielded no such effect. Notably, neither pan-HDACi nor HDAC3i reduced cytokine secretion in M2 macrophages. In contrast to previous reports in mouse macrophages, HDAC3i did not affect macrophage polarization in human cells. Likewise, HDAC3 was not required for IFNγ signaling or IFNβ secretion. Cytokine and gene expression analyses confirmed that IFNγ-treated macrophages consistently develop a cytokine response after LPS repeated challenge, but pretreatment with HDAC3i or *HDAC3* siRNA reinstates a state of tolerance reflected by general suppression of tolerizable genes, possibly through decreasing TLRs expression, and particularly TLR4/CD14. The development of endotoxin tolerance in macrophages is important to reduce exacerbated immune response and limit tissue damage. We conclude that HDAC3 is an attractive protein target to mediate macrophage reactivity and tolerance induction in inflammatory macrophages.

## Introduction

Histone acetylation controls chromatin remodeling, which in turn is thought to regulate gene transcription. Where histone acetyltransferases (HAT) add acetyl groups to lysine residues, thereby enabling transcription factor binding and subsequent gene expression, histone deacetylases (HDACs) remove histone residues leading to chromatin compaction, which generally results in gene repression (1, 2). Such epigenetic mechanisms involving HDACs have gained interest in immunology, as they were found to mediate innate immune-cell memory processes, as well as development of immune training and tolerance towards endotoxins (3–5). Notably, some reports found that the inhibition of HDACs by means of HDAC inhibitors (HDACi) ameliorated experimental colitis in mice (6, 7). Similar observations have since been made for human cells as well, where the non-selective broad-spectrum HDAC inhibitor Givinostat was found to confer anti-inflammatory properties in human pro-inflammatory macrophages (8, 9). However, clinical trials with pan-HDACi revealed multiple adverse events such as diarrhea, nausea and vomiting (10, 11). To reduce such side effects, HDAC-subtype-specific inhibitors with increased sensitivity and specificity have been developed.

In humans, the HDAC family consists of 18 members divided into class I HDACs (HDACs 1–3 and 8), class IIa HDACs (HDACs 4, 5, 7 and 9), class IIb HDACs (HDACs 6 and 10), class III sirtuins (Sirt1–7) and class IV HDACs (HDAC11) (12, 13). Although the members of each class show high structural similarity, genetic deletion of individual HDACs revealed that each HDAC has specific and unique roles with respect to substrate selectivity (14). Within the context of diseases, HDACs are reportedly important epigenetic regulators of the immune system (15). In rheumatoid arthritis for instance, the inhibition of class I/II or class III HDACs ameliorated clinical symptoms by blocking the production of IL-6 and TNFα (16). Similarly, deficiency of HDAC9 enhanced regulatory T cell numbers and ameliorated models of systemic lupus erythematosus and colitis (17). HDAC3 specifically was found to play a crucial role in orchestrating the inflammatory response of murine myeloid cells, such as dendritic cells (18–20) and macrophages (6, 21), by initiating transcription of inflammatory genes (12, 22) while at the same time limiting anti-inflammatory genes *in vivo* (23). In atherogenic macrophages, HDAC3 inhibition coaxed macrophage metabolism towards enhanced aerobic glycolysis, thereby protecting against apoptosis (24). Furthermore, myeloid cells obtained from *Hdac3* knockout mice stimulated anti-inflammatory wound healing, which was attributed to the loss of LPS-induced transforming growth factor β (TGFβ) (25).

Given these outcomes, several studies have hypothesized a potential role for HDAC3 in developing tolerance towards endotoxins, such as lipopolysaccharides (LPS) (5, 26, 27). The potential to develop LPS tolerance represents a state where immune cells mount a less pro-inflammatory response towards LPS upon subsequent treatments with it. Expectedly, LPS tolerance appears to be restricted to anti-inflammatory macrophages (M2) (28, 29) rather than interferon (IFN) γ-primed inflammatory macrophages (M1) in mouse and humans (30–34). Transcriptomic studies on mouse-derived bone marrow macrophages revealed that many of the TLR4-induced genes from both the MyD88-dependent and independent pathways were downregulated in LPS-tolerized macrophages and that the promoters of these downregulated genes often coincided with distinct histone modifications (26–28). Most studies however, were restricted to mouse cells and have not been translated to human.

In the current study, we investigated the effect of HDAC3i on differentiation and activation of IFNγ-primed human peripheral blood-derived monocytes and macrophages. To this end, we utilized an inhibitor targeting HDAC3 (HDAC3i; ITF3100) as well as the nonselective pan-HDACi (Givinostat; ITF2357) (12). As ITF3100 has residual activity towards HDAC6 (35), an inhibitor targeting HDAC6 (HDAC6i; ITF3107), was used a control. In contrast to studies on mice, we observe that macrophage differentiation and polarization is independent of HDAC3. Rather, we show that HDAC3 mediates pro-inflammatory cytokine production, potentially through its activity on transcription factors, thereby restricting induction of tolerance in inflammatory macrophages. Our data underscore the potential of HDAC3 to direct cytokine production and inflammation in human immune mediated inflammatory disease.

## Methods

### Reagents

Pan-HDAC inhibitor (Givinostat; ITF2357), HDACi with activity toward HDAC3 (and to a lesser extent, HDAC6) (HDAC3i; ITF3100) and HDAC6 alone (HDAC6i; ITF3107) were provided by Italpharmaco and used at concentrations described previously (12, 24). Anti-CD163-PE (BD Biosciences), anti-CD200R-Alexa Fluor 647 (Serotec), anti-CD80-PE (BD Biosciences), anti-CD284 (TLR4)-PE (BioLegend) and anti-CD14-PECy7 (Becton Dickinson) antibodies were used for flow cytometric analyses and anti-Histone H3 (Abcam), anti-Histone H3K18-Ac (Cell Signaling), anti-STAT1, anti-STAT1-p and anti-beta-actin (Santa Cruz) antibodies were used for western blot analysis.

### Monocyte Isolation and Cell Culture

Peripheral blood mononuclear cells (PBMCs) were obtained from whole blood of healthy donors (Sanquin Institute, Amsterdam, The Netherlands) by Ficoll density gradient (Invitrogen). The human biological samples were sourced ethically, and their research use was in accordance with the terms of informed consent under an IRB/EC approved protocol. Written informed consent was obtained from donors, as approved by the UK East of England—Cambridgeshire and Hertfordshire Research Ethics Committee and Amsterdam UMC Institutional Review Board under project number B07.002-X. CD14^+^ monocytes were positively selected from PBMCs using CD14 Microbeads according to the manufacturer’s instructions (Miltenyi Biotec).

To study the effect of HDACi on cytokine expression in monocytes and macrophages, CD14^+^ monocytes were used in subsequent analyses or polarized immediately after isolation towards M1 (classically activated) macrophages using 50 ng/mL IFNγ (R&D systems/Peprotech), or M2 (alternatively activated) macrophages using 40 ng/mL interleukin (IL) 4 (R&D systems/Peprotech) in culture media for 72 h. Freshly isolated CD14^+^ monocytes and M1- or M2-polarized macrophages were pre-treated with an increasing concentration (12, 37, 111, 333, and 1,000 nM) of HDAC3i (HDAC3 half maximal inhibitory concentration (IC_50_) = 144 nM; HDAC6 IC_50_ = 48 nM), HDAC6i (HDAC3 IC_50_ = 286 nM, HDAC6 IC_50_ = 2 nM), pan-HDACi (16, 36) or DMSO control for 30 min prior to stimulation with 100 ng/mL lipopolysaccharide (LPS) (Sigma) for 24 h.

To assess the effect of HDACi on monocyte polarization to the different macrophage phenotypes, freshly isolated CD14^+^ were incubated with either DMSO or 500 nM HDACi for 30 min prior to polarization to M0, M1 and M2 macrophages.

To investigate the endotoxin repeated challenge in M1 and M2 macrophages, M1 and M2 macrophages were initially treated with medium only or with 10 ng/mL LPS for 24 h, washed with PBS, and then challenged by treating the macrophages with medium only or with 100 ng/mL LPS for 3 h (to assess gene expression) to 4 h (to assess protein expression). Macrophages that were treated first with 24 h medium then challenged with medium are designated as M/M (medium/medium), macrophages stimulated with one dose of LPS after 24 h medium pretreatment are designated as M/L (medium/LPS), macrophages stimulated with LPS for 24 h and challenged with LPS are designated as L/L (LPS/LPS).

To study whether HDAC3 plays a role in regaining LPS tolerance in M1 macrophages, CD14^+^ monocytes were pretreated with either DMSO or HDAC3i for 30 min or with non-targeting scrambled siRNA or *HDAC3* siRNA for 48 h, after which they were polarized to M1 macrophages overnight with 50 ng/mL IFNγ and then M/M, M/L and L/L conditions were applied as described above.

Isocove’s Modified Dulbecco’s Medium (IMDM; Lonza) supplemented with 10% fetal bovine serum (FBS) (Lonza), 2 mM l-glutamine (Lonza), 100 U/ml penicillin (Lonza) and 100 U/ml streptomycin (Lonza) was used as culture media. 2x10^6^ cells were used for each condition in all experiments.

### siRNA-Mediated *HDAC3* Knockdown

The CD14^+^ monocytes were transfected with siGENOME human smartpool *HDAC3* siRNA or non-targeting scrambled siRNA for 48h with DharmaFECT™ transfection reagents according to manufacturer’s protocol (Dharmacon).

### Cytokine Expression Analysis

Secreted cytokine levels of IFNβ, tumor necrosis factor (TNF)α, IL-6, P40 and IL-10 were quantified in collected supernatant using the DuoSet^®^ ELISA Development Systems according the manufacturer’s protocol (R&D systems™).

### Isolation of Total RNA and Quantitative Reverse Transcriptase PCR

Total RNA was extracted using RNeasy Mini Kit (Qiagen) in accordance with the manufacturer’s instructions. Total RNA was transcribed into complementary DNA by qScript cDNA SuperMix (Quanta Biosciences) according to manufacturer’s instructions. Quantitative reverse transcriptase polymerase chain reaction (qPCR) was performed using a LightCycler^®^ FastStart DNA MasterPLUS SYBR Green I (Roche) on LightCycler 480 (Roche, Applied Science). Transcript expression levels were analyzed using LinRegPCR (37) and normalized to the geometric mean of three reference genes: *GAPDH, 36B4* and *HPRT*. Primer sequences used are listed in **Supplemental Table 1**.

### Western Blot Analysis

Cells were harvested and lysed in 50 μL ice-cold lysis buffer containing 150 mM NaCl, 0.5% Triton X-100, 5 mM Ethylenediaminetetraacetic acid (EDTA) and 0.1% SDS. Samples were mixed with sample buffer, denatured, and separated by SDS-PAGE before blotting onto polyvinyldifluoride membranes (Millipore). Membranes were blocked with non-fat dry milk (5% solution) and incubated overnight with the primary antibody. Membranes were then washed and incubated with HRP-conjugated secondary antibodies before visualizing the proteins using western blot substrate (Roche).

### Nuclear and Cytoplasmic Fraction Separation

Cells were collected, transferred to a pre-chilled collection tube, and washed twice with cold PBS. Cells were resuspended in 500 μL hypotonic buffer (Tris 20 mM pH 7.4, NaCl 10 mM, MgCl_2_ 3 mM), incubated for 15 min on ice, and 25 μL 10% NP40 was added and the solution was vortexed. The solution was then centrifuged for 10 min, and the supernatant was kept for analyses of the cytosolic fraction. The pellet was resuspended in 50 μL of cell extraction buffer (Tris 100 mM, NaCl 100 mM, Triton X100 1%, 1 mM EDTA, 10% glycerol, 1 mM egtazic acid (EGTA), 0.1% SDS, 0.5% deoxycholate, 20 mM Na_4_P_2_O_7_) and kept on ice for 30 min with 10 min vortex intervals. This fraction was then centrifuged for 30 min at 14,000xg and the supernatant was used for nuclear fraction analyses.

### Flow Cytometry

Cells were harvested and subsequently stained using the antibodies and dyes for the antigens of interest (CD200R, CD163 and CD80) before flow cytometric analysis using the LSRFortessa and FACSCalibur (both BD Biosciences). FlowJo (BD LSRFortessa™ cell analyzer) was used for data analysis.

### RNA-Sequencing

Total RNA was isolated from macrophages using the RNAeasy mini kit (Qiagen) and transcribed into cDNA by qScript cDNA SuperMix (Quanta Biosciences) according to manufacturer’s instructions. Subsequently, cDNA was sequenced in a 50 bp single-ended fashion on the Illumina HiSeq4000 to a depth of 35 million reads at the Amsterdam UMC Core Facility Genomics. Quality control of the reads was done with FastQC (v0.11.8) (38) and summarization through MultiQC (v1.0) (39). Raw reads were aligned to the human genome (GRCh38) using STAR (v2.7.0) (40) and annotated using the Ensembl v95 annotation (41). Post-alignment processing was performed through SAMtools (v1.9) (42), after which reads were counted using the featureCounts application (43) in the Subread package (v1.6.3) (44). Differential expression (DE) analysis was performed using DESeq2 (v1.22.2) (45) in the R statistical environment (v3.46.0) (46). The statistical model used in this analysis was expressed as: ∼*Donor* + *Treatment* + *Tolerized* + *Treatment*: *Tolerized*. Our research question specifically pertained the interaction between tolerization and HDAC3i treatment. Subsequent visualizations were done using ggplot2 (v3.2.0) (47). Enrichment analyses were performed using fgsea (v1.10.0) (48) and visualized using the GSEA plot function provided by Rodríguez-Córdoba (49).

### Public Data Analyses

The list of tolerizable and non-tolerizable genes was downloaded from the supplementary files published alongside Foster et al. (26) whereupon enrichment analyses were performed as described above. The transcription factors were obtained from the supplementary files published alongside Lambert et al. (50) and used to filter the differentially expressed genes for differentially expressed transcription factors. HDAC3 chromatin immunoprecipitation sequencing (ChIP-seq) peaks were obtained from untreated mice bone marrow derived macrophages (BMDMs) as stored in GSM2845618 and GSM2845619 from the dataset GSE106701 (51, 52). The ChIP-seq peak scores were log_2_ transformed and the mean was taken across samples. Gene symbols were translated between human and mice using the ortholog annotations available in biomaRt (v2.44.1) (53).

### Statistical Analyses of the FACS, qPCR, and ELISA Data

Data are shown as mean ± standard error of the mean (SEM) unless indicated differently. Statistical analysis was performed with GraphPad Prism v5.0a (GraphPad Software Inc.). For multi-experimental group analysis, data were subjected to one-way ANOVA or Student’s t-test followed by post-hoc test (Dunnett) for group differences. The two-tailed level of significance was set at p ≤ 0.05 (*), 0.01 (**) or 0.001 (***) for group differences.

## Results

### HDAC Activity Is Not Essential in Macrophage Polarization

**Figure 1:**
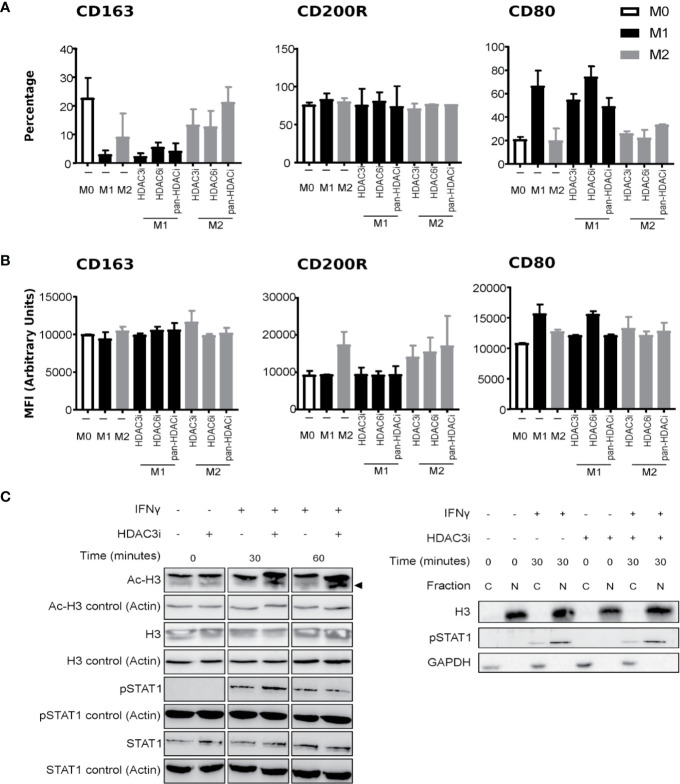
The effect of HDACi on macrophage polarization and IFNγ-induced STAT1 phosphorylation. Primary human CD14^+^ monocytes were treated with DMSO or 500 nM HDACi for 30 min prior to M0, M1 or M2 macrophages polarization, using media only, 50 ng/mL IFNγ or 40 ng/mL IL-4, respectively. Protein expression of macrophage markers CD163, CD200R and CD80 was assessed by FACS. **(A)** Data presented as percentage of CD163^+^, CD200R^+^ and CD80^+^ cells from total cells or **(B)** by mean fluorescence intensity (MFI) of CD163, CD200R and CD80 proteins (n=3). **(C)** Primary human CD14^+^ monocytes were polarized to M1 macrophages with 50 ng/mL IFNγ after which they were left untreated or treated with 500 nM HDAC3i for 0, 30 or 60 min, followed by a subsequent treatment with 50 ng/mL IFNγ for 24h. Total protein was assessed by Western blot for acetylated Histone H3 (“Ac-H3”) and non-acetylated Histone H3 (“H3”), phosphorylated STAT1 (“P-STAT1”) and non-phosphorylated STAT1 (“STAT1”) (left). Actin-α (“Actin”) was used as loading control. Protein levels were assessed for phosphorylated STAT1, and Histone H3 in the cytoplasmic (“C”) and nuclear (“N”) fractions (right). GAPDH was used as loading control.

To understand endogenous HDAC expression, we quantified the basal levels of HDACs in polarized macrophages. To this end, human primary CD14^+^ monocytes were polarized into M1 and M2 macrophages using IFNγ and IL-4, respectively, or left untreated in medium (M0 macrophages). We verified the polarization by measuring the gene expression of *CD163*, *CD64*, and *CD200R*, that mark M0, M1, and M2 phenotypes, respectively (54–58) (**Supplemental Figure 1A**). In addition, we investigated the cytokines typically secreted by M1 (TNFα and IL-6), or M2 (IL-10) macrophages (**Supplemental Figure 1B**). Comparison of the HDACs between the macrophage subsets suggested that HDAC expression did not significantly differ (**Supplemental Figure 2**). As previous reports demonstrated that the loss of HDAC3 augmented M2 polarization in mouse macrophages (23), we focused on HDAC3 to investigate whether a similar mechanism would be in place for human macrophages. To this end, we treated primary human CD14^+^ monocytes with Control (DMSO) or with 500 nM of pan-HDACi, HDAC3i, or HDAC6i (36) prior to polarization to M0, M1 and M2 macrophages as described above. We then investigated the effect of HDACi on the polarization markers by flow cytometry. We specifically studied the macrophage subset markers CD163, CD200R and CD80 to distinguish macrophage polarization. Pre-treating CD14^+^ monocytes with HDAC3i, HDAC6i or pan-HDACi did not significantly alter the polarization markers of macrophages derived from those monocytes. However, non-statistically significant downregulation in CD80^+^ cell frequency and CD80 protein surface expression in HDAC3i and pan-HDACi treated cells was observed (**Figures 1A, B**). This data suggests that HDACs may be redundant in the polarization of human M1 or M2 macrophages.

We next addressed whether HDAC3 affected M1 activity through intervening with the polarization process and IFNγ signaling (**Figure 1C**). First, we validated that HDAC3i effectively enhanced acetylation at histone H3 at the concentration used both in the presence and absence of IFNγ. Treatment of the M1 macrophages with HDAC3i displayed no interference in IFNγ-induced STAT1 phosphorylation, nor did it affect STAT1 translocation into the nucleus (**Figure 1C**). Together, our results demonstrate that HDAC3i does not modify human macrophage polarization or the potential of IFNγ to prime cells, as suggested for mouse cells (25).

### HDAC3 Inhibition Suppresses LPS Induced Cytokine Secretion in Monocytes and M1 Macrophages but Not in M2 Macrophages

**Figure 2:**
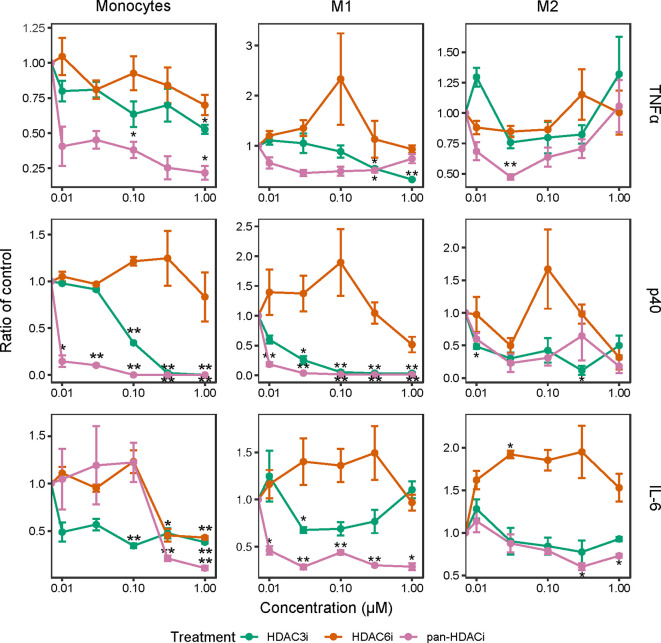
Cytokine production over increasing HDACi dosage in LPS-stimulated macrophages. Freshly isolated primary human CD14^+^ monocytes and polarized M1 and M2 macrophages were treated with control DMSO or an increasing concentration of HDACi (12, 37, 111, 333 and 1000 nM) for 30 min. The cells were then stimulated with 100 ng/mL LPS for 24h. Protein levels of pro-inflammatory cytokines TNFα and IL-6, as well as p40 in the supernatant were measured by ELISA. The y-axis indicates the fold change in cytokine protein expression relative to DMSO control. From left to right: Monocytes, M1 macrophages and M2 macrophages. *P < 0.05; **P < 0.01 (n=3).

We next investigated whether the inhibition of HDACs reduced the release of inflammatory cytokines in freshly isolated CD14^+^ monocytes as well as in M1 and M2 polarized macrophages. In LPS-stimulated monocytes and M1 macrophages, pretreatment with pan-HDACi and HDAC3i, but not HDAC6i, led to a dose-dependent decrease of TNFα, p40 and IL-6 cytokine secretion (**Figure 2**). The effects of HDAC inhibition on cytokine secretion were observed to a lesser extent in M2 macrophages (**Figure 2**). Overall, secretion of TNFα and p40 was not altered in M2 macrophages treated with pan-HDACi, HDAC3i or HDAC6i. These results demonstrate a prominent role for HDAC3 in LPS stimulated human monocytes and inflammatory M1 macrophages in the expression of inflammatory cytokines.

### HDAC3 Blocks the Induction of LPS-Tolerance in Inflammatory Activated M1 Macrophages

**Figure 3:**
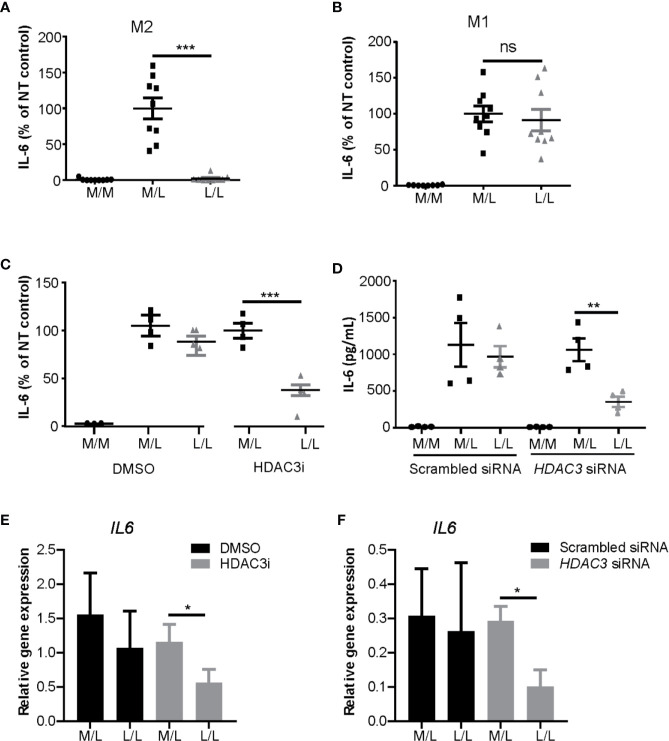
HDAC3i and *HDAC3* siRNA restored the impaired LPS-tolerance in M1 macrophages. Primary human CD14^+^ monocytes were polarized to M1 or M2 macrophages with IFNγ or IL-4 for overnight, respectively, and then were tolerized (“L/L”) with 10 ng/mL LPS or were left non-tolerized (“M/L”) for 24 h after which they were exposed to 100 ng/mL LPS for 4 h. As a control, LPS-naïve sample was included as well (“M/M”). **(A)** Protein level of IL-6 in the supernatant was measured by ELISA in M2 (n=9) or in **(B)** M1 macrophages (n=9). **(C)** Primary human CD14^+^ monocytes were pretreated with DMSO control or 500 nM HDAC3i 30 min prior to polarization with IFNγ. The cells were then “L/L” or “M/L”. IL-6 protein level was measured by ELISA (n=4). **(D)** Primary human CD14^+^ monocytes were pretreated with scrambled siRNA control or *HDAC3* siRNA 48h prior to polarization with IFNγ. The cells were then “L/L” or “M/L”. IL-6 protein level was measured by ELISA (n=4). **(E)** Relative gene expression of *IL6* in DMSO- or HDAC3i-pretreated “M/L” and “L/L” macrophages (prepared as described above) (n=3). **(F)** Relative gene expression of *IL6* in scrambled siRNA- or *HDAC3* siRNA-pretreated NT and T macrophages (prepared as described above) (n=3). *P < 0.05; **P < 0.01; ***P < 0.001; n.s.: not significant.

Having observed that HDAC inhibition decreased cytokine expression in M1 macrophages, we next addressed the role of HDAC3 in repeated LPS challenge protocol. M1 and M2 polarized macrophages were stimulated with a low concentration of LPS (10 ng/mL) overnight to induce LPS tolerance state, after which they were washed and re-stimulated with a higher concentration of LPS (100 ng/mL) (28, 59). As expected, we observed reduction of IL-6 protein expression in L/L M2 macrophages (**Figure 3A**), but not in L/L M1 macrophages (**Figure 3B**). Notably however, treatment of monocytes with HDAC3i prior to M1 polarization allowed for these cells to become tolerant, albeit not to the same extent as observed for L/L M2 macrophages (**Figure 3C**). To validate the previous observations, we performed siRNA-mediated knock-down to reduce *HDAC3* expression prior to polarization into M1 macrophages, achieving approximately 80% reduction in *HDAC3* expression (**Supplemental Figure 4A**). *HDAC3* knock-down yielded a comparable protein (**Figures 3C, D**) and mRNA expression reduction (**Figures 3E, F**) of IL6, confirming our previous observations.

To investigate this further, we tested whether HDAC3i reinstated tolerance by reducing IFNβ secretion as was observed in IFNγ-primed mouse macrophages (22, 27). However, when we measured the secretion of IFNβ in M1 macrophages derived from monocytes pretreated with HDAC3i, an elevated rather than a reduced IFNβ level was observed (**Supplemental Figure 3A**). Furthermore, the potential of IFNβ to activate STAT1 phosphorylation was unaffected by any of the HDACis tested (**Supplemental Figures 3B, C****)**. Taken together, HDAC3i overcomes the potential of IFNγ to block tolerance induction, *via* a mechanism independent of enhanced IFNγ or IFNβ production nor signaling.

### HDAC3 Inhibition Causes Upregulation of Gene Expression in LPS-Naïve M1 Macrophages

**Figure 4:**
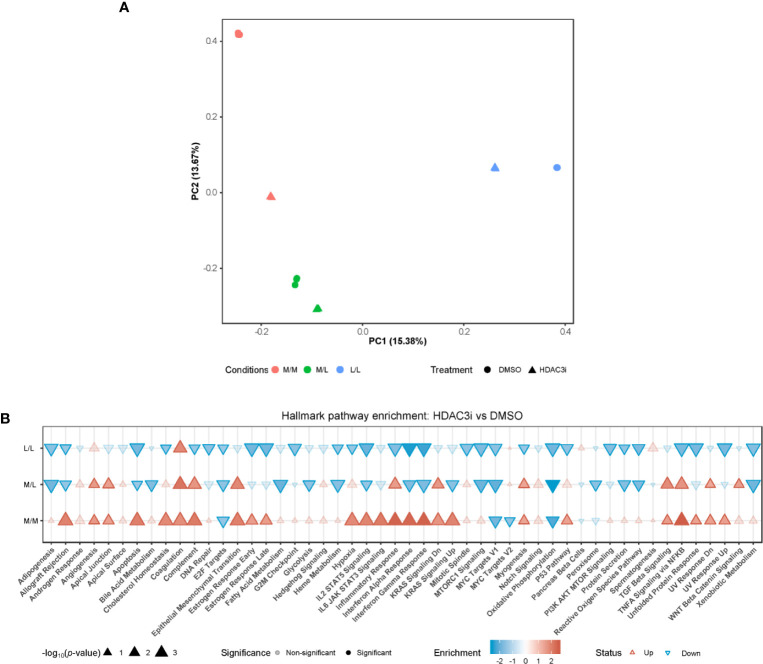
Monocytes pretreatment with HDAC3i induced transcriptional changes of M1 macrophages. Primary human CD14^+^ monocytes were treated with 500 nM of HDAC3i or DMSO prior to polarization with IFNγ overnight yielding M1 macrophages. The macrophages were then washed and exposed to 3 conditions: non-treated with LPS naïve (“M/M”), non-tolerized (“M/L”) or tolerized (“L/L”). RNA was isolated from the macrophages 3 h after last LPS stimulation (n=3). **(A)** Visualization of the first (“PC1”) and second (“PC2”) principal components calculated from the RNA-sequencing data annotated with the percentage explained variance. **(B)** Hallmark enrichment analysis when comparing HDAC3i with DMSO-treated cells for the different tolerization states. The direction and color of the arrow indicates the direction and size of the enrichment score, the size of the arrow represents is proportional to the –log_10_(*p*-value), and non-transparent arrows represents significantly affected pathways.

To address the pathways by which HDAC3i overcomes resistance to tolerance induction in M1 macrophages, we profiled the transcriptome through RNA-sequencing. Freshly isolated CD14^+^ monocytes were treated with HDAC3i or DMSO as a control prior to polarization to M1 macrophages using IFNγ and then treated as naïve control without any LPS stimulation (M/M), LPS repeated challenge (L/L) or only treated once with LPS (M/L) after which the transcriptome was profiled at the same time-point for all groups. Principal component (PC) analysis revealed that the first principal component (PC1) separated the M/M, M/L and L/L macrophages treated with DMSO (**Figure 4A**). Surprisingly, the treatment of HDAC3i had a profound effect on M/M macrophages (**Figure 4A**). Little separation was observed for M/L macrophages between HDAC3i- and DMSO-treated, whilst HDAC3i largely affected gene expression in L/L macrophages (**Figure 4A**). To better understand the effect of HDAC3i treatment on the transcriptome, we compared HDAC3i- with DMSO-treated macrophages across the different tolerization states (**Figure 4B**). When investigating the associated pathways, we found that many of the hallmark pathways that were upregulated in HDAC3i-treated M/M macrophages, were downregulated in M/L and L/L groups (**Figure 4B**). Our results demonstrate that while HDAC3i upregulates multiple inflammatory pathways in M/M macrophages, such inflammatory processes are subsequently downregulated in the presence of LPS.

### Pretreatment With HDAC3i Partially Recovers Tolerance to LPS in M1 Macrophages

**Figure 5:**
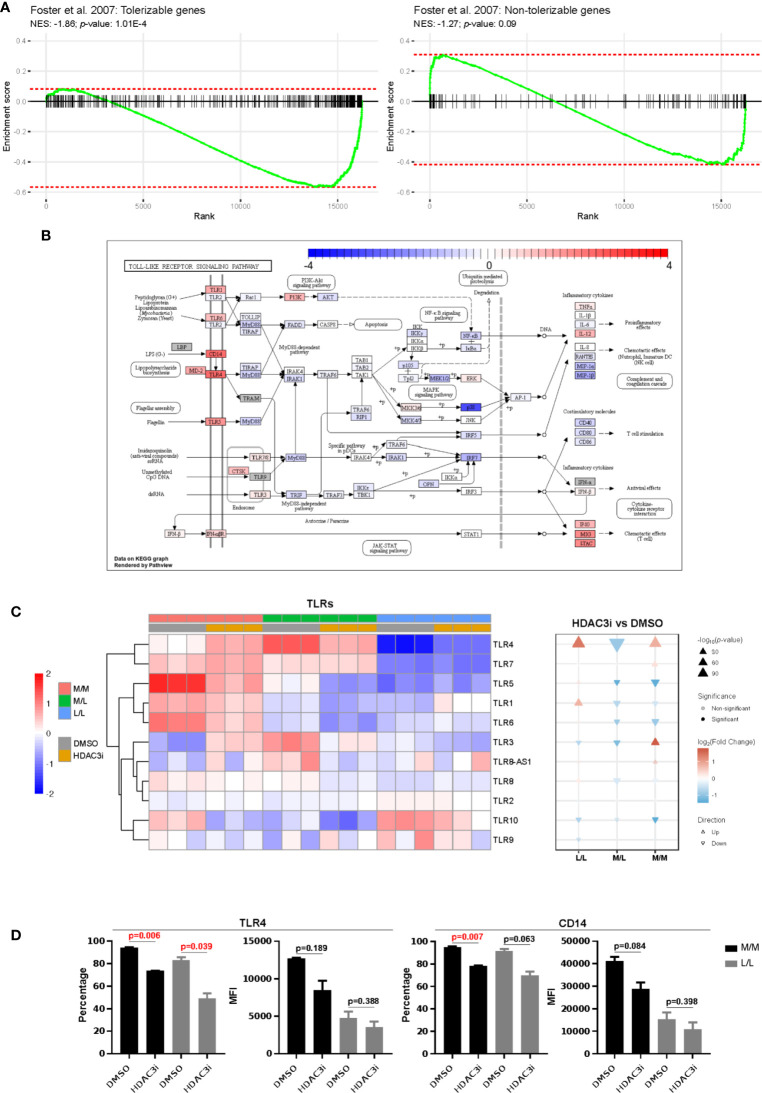
Comparison of the tolerized versus non-tolerized M1 macrophages derived from HDAC3i-pretreated CD14^+^ monocytes with the tolerized versus non-tolerized M1 macrophages derived from HDAC3i-pretreated CD14^+^ monocytes. An interaction analysis was performed to identify genes that displayed differential expression when comparing tolerized with non-tolerized for M1 macrophages derived from DMSO- or HDAC3i-treated primary human CD14^+^ monocytes (n=3). **(A)** Gene set enrichment analysis performed on the tolerizable and non-tolerizable gene sets as defined by Foster et al. in mice. **(B)** The KEGG Toll like receptor signaling pathway with colors representing the effect size obtained from interaction analysis. **(C)** Heatmap and gene set enrichment analysis of TLRs in “M/M”, “M/L” and “L/L” macrophages. **(D)** FACS analysis of TLR4 and CD14 in “N” and “NT” M1 macrophages derived from DMSO-treated or HDAC3i-treated primary human CD14^+^ monocytes. Data is presented as percentage or mean of florescence intensity (n=2).

We next compared the differences in gene expression between HDAC3i and DMSO treatment upon repeated challenge with LPS by means of an interaction analysis. We observed the difference of differences to be statistically significant for 5,621 genes, where a majority (3,451) were downregulated in HDAC3i-treated L/L versus M/L macrophages relative to DMSO-treated L/L versus M/L macrophages (**Supplemental Table 2**). Enrichment analysis on gene sets defined as tolerizable and non-tolerizable by Foster *et al*. for macrophages in mice (26) indicated that the majority of the tolerizable genes were downregulated in the HDAC3i pretreated macrophages, whereas the non-tolerizable genes were seemingly less affected (**Figure 5A**). Among the tolerizable genes we observed that the TLR pathway in particular displayed an interesting pattern of expression. Whereas genes encoding proteins downstream of the TLRs, such as *IL6*, *RANTES* (*CCL5*), *IL1β, MIP-1α* and *MIP-1β* were downregulated, *CD14* as well as the *TLR* genes themselves were upregulated (**Figure 5B**). Comparing HDAC3i- with DMSO-treated macrophages indicated that most *TLRs*, and in particular *TLR4*, were upregulated for M/M and L/L macrophages (**Figures 5B, C**). Pretreatment with HDAC3i or *HDAC3* siRNA enhanced *TLR4* gene expression in M/M and L/L macrophages (**Figure 5C** **and** **Supplemental Figure 4B**). We therefore investigated protein expression of TLR4 alongside CD14 by flow cytometry. In contrast to mRNA, cell-surface expression of TLR4 and CD14 were found to be downregulated in HDAC3i pretreated cells (**Figure 5D**). Together, our data therefore shows that HDAC3i contributes to LPS-tolerance through downregulation of gene sets referred to as “tolerizable” including those belonging to the TLR pathway.

### HDAC3 Inhibition Potentially Abrogates HDAC3 From Binding Tolerizable Transcription Factors

**Figure 6:**
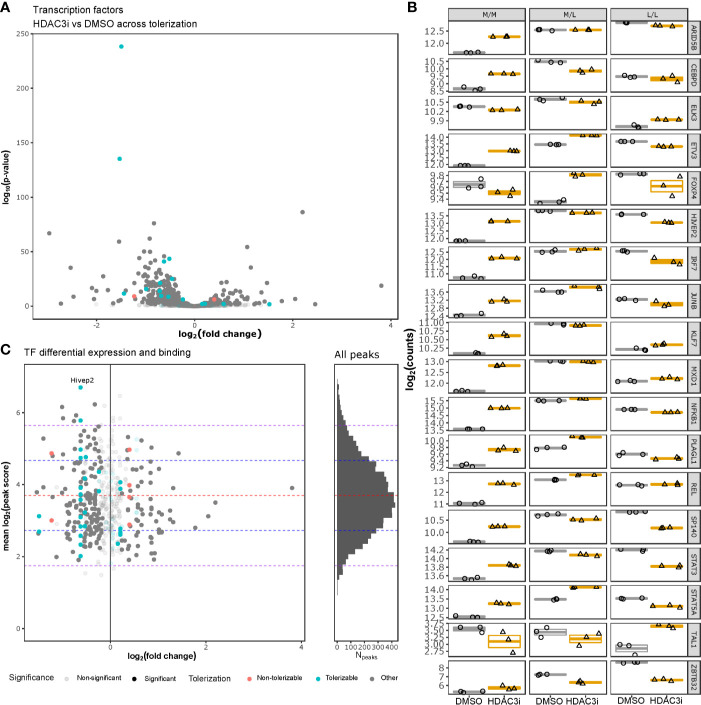
HDAC3i-treatment effect on transcription factors. Gene expression of human tolerizable TFs across the non-tolerized and tolerized states was interrogated and compared with HADC3 binding in mice macrophages. **(A)** Gene expression of tolerizable human TFs, obtained from Foster et al., 2007 and Lambert et al., 2018, respectively, were presented as a volcano plot with the log_2_ fold-change on the x-axis and the –log_10_(*p*-value) on the y-axis. **(B)** The expression in log_2_(counts) on the y-axis of the significantly differentially expressed TFs (*ARID5B, CEBPD, ELK3, ETV3, FOXP4, HIVEP2, IRF7, JUNB, KLF7, MXD1, NFKB1, PLAGL1, REL, SP140, STAT3, STAT5A, TAL1 and ZBTB32*) for M/M, M/L and L/L macrophages, pretreated either with DMSO or HDAC3i. **(C)** Overlap of the differentially expressed tolerizable TFs with the HDAC3-bound TFs in mouse BMDMs as obtained from GSE106701 samples GSM2845618 and GSM2845619. The x-axis represents the log_2_(fold change), while the y-axis represents the mean log_2_(MACS peak score). Histogram on the right represents the mean log_2_(MACS peak score) distribution (y-axis) for all the HDAC3-binding regions reported by Czimmerer et al. x-axis represents number of peaks (N_peaks_). The red, blue and purple dashed horizontal lines represent the mean, first and second standard deviations, respectively.

To understand whether HDAC3i affected global transcription, we specifically investigated how transcription factors were affected by HDAC3i in both the L/L and M/L macrophages. Interrogating the DEGs for 1639 curated human transcription factors (TFs) (50) indicated that 388 TFs were significantly differentially expressed (**Figure 6A** **and** **Supplemental Table 3**). Notably, the majority of the TFs (302 versus 86) were significantly more downregulated in the L/L macrophages relative to the M/L macrophages (**Figure 6B**). Overlapping the differentially expressed TFs with the tolerizable genes (26) indicated that HDAC3i resulted in an initial upregulation of *ETV3*, *STAT5A*, and *NFKB1* in both M/M and M/L macrophages, but a significant downregulation in the L/L macrophages, which in the case of *STAT5A* and *NFKB1* resulted in a lower expression relative to M/M macrophages. Taken together, our results suggest that the ameliorative effect of HDAC3 inhibition only occurs after the secondary LPS stimulation.

To understand whether HDAC3 directly affects transcription of TFs, we analyzed ChIP-sequencing data on HDAC3 binding in mouse M-CSF differentiated bone marrow derived macrophages (BMDMs) obtained from GSE106701 (51, 52). Combining the reported peaks from samples GSM2845618 and GSM2845619 with the differential expression analysis on TFs indicated that mouse orthologs of the differentially expressed TF genes *HIVEP2*, *ELK3*, *IRF7*, *NFKB1*, *ARID5B*, *REL*, *KLF7*, *ETV3* and *MXD1* were bound by HDAC3, with one peak associated to the intergenic region of *HIVEP2* belonging to the top 2.5% strongest peaks (**Figure 6C** and **Supplemental Table 4**). Taken together, many differentially expressed TFs appear subject to HDAC3 activity, and could therefore explain the functional effects of HDAC3i. However, further analyses of HDAC3 DNA interaction did not reveal direct promotor binding of these affected TF (**Supplemental Figure 5**) (51, 52), suggesting that HDAC3i likely affects TF gene expression indirectly.

## Discussion

This study suggests that HDAC3 is required for the organization of the inflammatory gene program and supporting mechanisms to evade tolerance induction in such a way that we are capable of partially recapitulating tolerance towards LPS in M1 macrophages by treatment with HDAC3i. We showed that HDAC3i resulted in the reduced expression of LPS-induced pro-inflammatory cytokines after a second exposure to LPS in human monocytes and M1 macrophages, but not in M2 macrophages, implying that the M1 macrophages have developed some degree of tolerance towards LPS.

Through RNA-sequencing, we found that HDAC3i-pretreated macrophages without any LPS exposure upregulate most of the Hallmark gene sets, inflammatory pathways included. We are unsure why this is the case. While searching for transcription factors bound by HDAC3, we observed in the data published by Czimmerer et al. that HDAC3 bound upstream of the IκBα-encoding genes *Nfkbiz* and *Nfkbia* in mice, both of which were also upregulated in HDAC3i pre-treated macrophages (**Supplemental Figures 6A, B**) (51, 52). As IκBα protein prevents NFκB nuclear translocation and gene expression in both in human and mice, we speculate that this mechanism explains how HDAC3 negatively regulates NFκB activity and that HDAC3i reduces expression of NFkB dependent pro-inflammatory genes as measured in this study. Despite this initial pro-inflammatory phenotype, HDAC3i ameliorates the pro-inflammatory phenotype after a second tolerization exposure to LPS, which in the down-regulation of most Hallmark gene sets. Further investigation into LPS revealed that tolerizable genes, as defined by Foster *et al*. were notably more downregulated in the HDAC3i pretreated M1 macrophages compared to the DMSO-pretreated M1 macrophages. By carefully investigating the LPS-induced TLR signaling pathway, we observed that most of the downstream genes were downregulated. Akin to the observations made by Porta et al., we observed that HDAC3i resulted in the downregulation of genes located in both the MyD88-dependent and MyD88-independent pathways (59). However, not all genes belonging to the TLR signaling pathway are downregulated (26, 27, 60). In fact, we observed that in a tolerant state relative to the non-tolerant state, many TLR encoding genes were more upregulated with HDAC3i pretreatment, albeit protein levels were decreased. An explanation for this may be that HDAC3 regulates inflammatory gene expression indirectly through processes that mediate mRNA stability (16). Alternatively, we hypothesize that HDAC3i stimulates endocytosis of TLR4 and CD14 (59) given that HDAC3i significantly enhances the expression of genes involved in facilitating the TLR4 endocytosis in tolerized versus non-tolerized macrophages, such as *CTNND1* or *P120* (61), *GMFG* (60), and affecting some genes that have a role in CD14 endocytosis such as *TRPM7* (61) (**Supplemental Table 2**).

Despite our observations, there are multiple considerations. First, the HDAC3i compound used, ITF3100, also displays a weak affinity to HDAC6 (12, 24). However, at the concentrations we used, ITF3100 was found to be selective for HDAC3 (12, 24). Moreover, equimolar use of a specific HDAC6i did not affect cytokine expression. Furthermore, silencing *HDAC3i* through siRNA resulted in the same reduction in pro-inflammatory cytokine expression as was seen when macrophages were pretreated with HDAC3i. Second, we have not been able to show the direct mechanism by which HDAC3 interferes with tolerance induction. HDAC3 is known to form a super complex with the NFκB subunit p50 NCor (NCoR-p50) complex in tolerized cells (22). Previous studies in mice revealed that a double knockout for the NFκB subunit p50 gene resulted in peritoneal macrophages that were unable to induce tolerance (59, 60). HDAC3-mediated deacetylation of p50 by HDAC3 is thought to be critical in tolerance induction and blocking transcription of tolerizable genes (27). IFNγ overcomes the repressive activity of p50 through Bcl3 (28), resulting in the absence of tolerance in M1 macrophages. It was suggested that IFNγ represses p50 and maintains IFNβ levels such that tolerizable genes remain active even after repeated exposures to LPS in mice. Indeed, p50^-/-^ mice macrophages were found to display high levels of IFNβ (38). However, we show that the secretion of IFNβ in HDAC3i treated cells was elevated rather than reduced. Moreover, the potential of IFNβ to activate STAT1 through phosphorylation remains unaffected under HDAC3i, making the involvement of altered IFNβ production in the induction of tolerance less likely in human cells. However, further mechanistic studies are necessary that investigate the chromatin structure of STAT1-binding in the presence and absence of HDAC3i.

Third, the exact protocols of inducing LPS tolerance in macrophages remains a point of discussion (62). While most studies appear to agree on an initial low concentration of LPS, followed by second stimulation with a higher concentration of LPS, the exact time between both stimulations differs. Where we opted for 24 h stimulation with LPS (26, 63–66), some studies have opted using a 16 h for the initial LPS treatment time (67), whereas others used a 48 hour initial LPS-treatment to ensure that the residual activity of LPS-induced genes is minimal (64).

Overall, our data suggest that HDAC3 acts as an epigenetic brake on the induction of LPS tolerance. This observation implicates HDAC3 as a potentially important anti-cytokine target in inflammatory macrophages, the dysregulation of which has been implicated in inflammatory disease, such as inflammatory bowel disease (68) and rheumatoid arthritis (12). For instance, IFNγ may antagonize anergy in mucosal macrophages (69), a process potentially abrogated by HDAC3i. Our findings may therefore be important in establishing HDAC3 as therapeutic target to reduce macrophage cytokine production in the inflamed tissues and inflammatory insults in immune disorders.

## Data Availability Statement

The datasets presented in this study can be found in online repositories. The names of the repository/repositories and accession number(s) can be found in the article/**Supplementary Material**.

## Author Contributions

Study design and conduction of the study, laboratory and writing of the manuscript: MG, JZ, and AL. Bioinformatics analyses: MG and AL. Study design, supervision and writing of the manuscript: NH, DT, PH, MW, WJ. Laboratory analyses: RS, JD, CV, TM, IB. Technical support: PPH. All authors contributed to the article and approved the submitted version.

## Funding

MG and AL are funded by European Union’s Horizon 2020 research and innovation program under Grant Agreement No. ITN-2014-EID-641665. This project has also received funding from the European Union’s Horizon 2020 research and innovation programme under the Marie Skłodowska-Curie grant agreement No 814168. WJ is funded through the Dutch Ministery of Economic Affairs (TKI-Health Holland). GlaxoSmithKline provided support in the form of salary for authors AL, NH, and DT. GlaxoSmithKline had no additional role in the study design, data collection and analysis, decision to publish, or preparation of the manuscript. Funding for open access charge will be covered by Amsterdam University Medical Centers, University of Amsterdam.

## Conflict of Interest

MG, AL, NH, PH and DT were employed by GlaxoSmithKline.

The remaining authors declare that the research was conducted in the absence of any commercial or financial relationships that could be construed as a potential conflict of interest.
