# Supplementary material for: HDAC3 Mediates the Inflammatory Response and LPS Tolerance in Human Monocytes and Macrophages
Source: Front Immunol. 2020 Oct 5;11:550769. doi: 10.3389/fimmu.2020.550769 (PMC7573361; doi:10.3389/fimmu.2020.550769)
Supplement: Supplementary file 1 [file DataSheet_1.docx]

**Supplemental Figures**

**Supplemental Figure** **1**. Expression of M0, M1 and M2 macrophage markers. Freshly isolated human CD14^+^ monocytes from buffy coats were polarized into M1 macrophages using 50 ng/mL IFNγ, M2 macrophages using 40 ng/mL IL-4 or M0 macrophages using only culture media for 72 hours. M0, M1 and M2 macrophages are characterized by (A) the gene expression of surface markers CD163, CD64 and CD200R and (B) and by ELISA of inflammatory cytokines markers: TNFα and IL-6, as well as anti-inflammatory marker IL-10. Gene expression was relative to the geometric mean of 3 reference genes: HPRT, 36B4 and GAPDH. CD200R relative gene expression in M1 and M2 was presented as fold change relative to M0 macrophages. Statistical significance was calculated through an ANOVA F-test (n=3).

Supplemental Figure 2. Expression of HDACs in M0, M1 and M2 macrophages. Gene expression for HDACs: HDAC1, HDAC2, HDAC3, HDAC4, HDAC5, HDAC6, HDAC7, HDAC8, HDAC9, HDAC10, HDAC11 and SIRT1 in M0, M1 and M2 macrophages derived from primary human CD14^+^ monocytes (n=3). Expression levels were normalized to geometric mean of three reference genes: TBP, 36B4 and GAPDH. Statistical significance was calculated through an ANOVA F-test.

**Supplemental** Figure 3. Protein expression of IFNβ in **M/M**, **L/L** and **M/L** M1 macrophages **derived from DMSO-treated or HDAC3i-treated primary human CD14^+^ monocytes**. (A) Protein concentration of IFNβ for samples non-treated with LPS naïve (M/M), treated once with LPS (M/L) and LPS challenged (L/L) M1 macrophages derived from DMSO-treated or HDAC3i-treated primary human CD14^+^ monocytes (n=4). (B) M1 macrophages were treated for 30 minutes with pan-HDACi, HDAC3i or HDAC6i whereupon they were stimulated with 100 U/ml IFNβ for 60 minutes. STAT1 and STAT1 phosphorylation were assessed by Western blot. (C) Quantification of the western blot where the results represent the mean and standard error of the mean (SEM) of 3 separate experiments.

**Supplemental** Figure 4. Treatment with *HDAC3* siRNA prior to M1 polarization, increases TLR4 gene expression in M/M and L/L macrophages. (A) Primary human CD14^+^ monocytes were pretreated with scrambled siRNA control or *HDAC3* siRNA for 48h. The cells were then treated with IFNγ for 4h and gene expression of *HDAC3* was measured by qPCR (n=3). (B) Primary human CD14^+^ monocytes were pretreated with scrambled siRNA control or *HDAC3* siRNA 48h prior to overnight polarization with IFNγ. The cells were then M/M or L/L. TLR4 gene expession was mueasured by qPCR (n=3).

**Supplemental** Figure 5. HDAC3 binds the differentially expressed TFs. Visualization of HDAC3-binding regions (black bars) relative to the genomic location (mm10) for the differentially expressed TFs in mouse BMDMs. The first track represents the location of the transcripts (*X-axis*) whereas the second and third track represent the location and log_2_(MACS peak score) from GSM2845618 and GSM2845619, respectively (*Y-axis*). Bars represent the size of the regions occupied by HDAC3 as well as the binding intensity as reported by *Czimmerer et al.*

**Supplemental** Figure 6. Chromatin binding and gene expression of Nfkbia and Nfkbiz. (A) Visualization of HDAC3-binding regions relative to the genomic location (mm10) for *Nfkbia* and *Nfkbiz* in mouse BMDMs. The first track represents the location of the transcripts whereas the second and third track represent the location and log_2_(MACS peak score) from GSM2845618 and GSM2845619, respectively. Bars represent the size of the regions occupied by HDAC3 as well as the the binding intensity as reported by *Czimmerer et al.* (B) The expression in log_2_(counts) on the y-axis of *NFKBIA* and *NFKBIZ* for M/M, M/L and L/L macrophages, pretreated either with DMSO or HDAC3i.

**Supplemental Table 1. Primer sequences.** Primer sequences used in the quantitative PCR analysis of the genes of interest.

| **Gene** | **Forward (5’-3’)** | **Reverse (5’-3’)** |  |
| --- | --- | --- | --- |
| GAPDH | GTCAGTGGTGGACCTGACCT | TGAGCTTGACAAAGTGGTCG |  |
| HPRT | AGTTCTGTGGCCATCTGCTT | GTTAAACAACAATCCGCCCA |  |
| 36B4 | TCATCAACGGGTACAAACGA | GCCTTGACCTTTTCAGCAAG |  |
| CD64 | GCAGGAACACATCCTCTGAA | GTAACTGGAGGCCAAGCACT |  |
| CD200R | GAGCAATGGCACAGTGACTGTT | GTGGCAGGTCACGGTAGACA |  |
| CD163 | ACATAGATCATGCATCTGTCATTTG | ATTCTCCTTGGAATCTCACTTCTA |  |
| HDAC1 | CTTCCTGCTGAGTCCCTCAC | GGCACCCTTTATGGTTCAAA |  |
| HDAC2 | AGGCCCCATAAAGCCACTGCC | AGCTCCAGCAACTGAACCGCC |  |
| HDAC3 | CTGTGTAACGCGAGCAGAAC | GCAAGGCTTCACCAAGAGTC |  |
| HDAC4 | GACGGTGCACTCGGAAGCCC | CTACCACGCAGCCCACAGCC |  |
| HDAC5 | GTGACACCGTGTGGAATGAG | AGTCCACGATGAGGACCTTG |  |
| HDAC6 | GGGTGCCAGCAGCCAGATCG | AGCAGGTGGGTGAGGTGGGC |  |
| HDAC7 | GTCCTGGTGTCTGCTGGATT | AAGGGGATCCACCCTGTTAC |  |
| HDAC8 | GCGTGATTTCCAGCACATAA | ATACTTGACCGGGGTCATCC |  |
| HDAC9 | GCCCACAGGAACTTCTGACT | GAACTCTAAGCCAGATGGGG |  |
| HDAC10 | TCCACCCGAGTACCTTTCAC | GATCCTGTGTAGCCCGTGTT |  |
| HDAC11 | CGAGGCACCTAACATCCATT | TGCGCTACAAGAACTTTCCA |  |
| SIRT1 | TCAGTGGCTGGAACAGTGAG | TCTGGCATGTCCCACTATCA |  |
| IL6 | AGTGAGGAACAAGCCAGAGC | GTCAGGGGTGGTTATTGCAT |  |
| TLR4 | CGGTGATAGCGAGCCACGCATT | ATATTAGGAACCACCTCCACGCAGGG |  |

**Supplemental Table 2. Genes whose expression displays an interaction between HDAC3i treatment and tolerization.** Interaction analysis comparing the difference HDAC3i and DMSO with the difference between tolerized and non-tolerized. Output as obtained from DESeq2 ranked by the *p-*value (n=3). Columns represent the Ensembl gene ID, the basal mean count, the log_2_ fold change, the standard error of the log_2_ fold change, the Wald statistic, the p-value, the Benjamini-Hochberg adjusted p-value, and the HGNC symbol.

**Supplemental Table 3. Transcription factors whose expression displays an interaction between HDAC3i treatment and tolerization.** A subset of the interaction analysis comparing the difference HDAC3i and DMSO with the difference between tolerized and non-tolerized (Supplemental Table 2) representing the transcription factors as obtained from *Lambert et al.*. Output as obtained from DESeq2 ranked by the *p-*value (n=3). Columns represent the Ensembl gene ID, the basal mean count, the log_2_ fold change, the standard error of the log_2_ fold change, the Wald statistic, the p-value, the Benjamini-Hochberg adjusted p-value, and the HGNC symbol.

**Supplemental Table 4. HDAC3-bound tolerizable transcription factors.** The differentially expressed transcription factors as found in the interaction analysis were interrogated for HDAC3-binding in mice BMDMs (GSM2845618 and GSM2845619) as obtained from *Czimmerer* *et al.* (GSE106701). Columns A to F represent annotations of the reported HDAC3-binding regions, corresponding to human and mouse Ensembl Gene IDs alongside the HGNC/MGI gene symbols, the provided annotation by Czimmerer *et al.*, and their tolerizable status as reported by Foster *et al.*. Columns G to K represent statistics obtained from the ChIP-seq experiment reported by *Czimmerer et al.*, corresponding to the untransformed and log_2_-transformed MACS peak scores for GSM2845618 and GSM2845619, and the mean of the log_2_-transformed MACS peak scores. Columns L to Q represent the output as obtained from DESeq2 with columns corresponding to the basal mean count, the log_2_ fold change, the standard error of the log_2_ fold change, the Wald statistic, the p-value, and the Benjamini-Hochberg adjusted p-value.
