# Supplementary material for: HDAC3 Mediates the Inflammatory Response and LPS Tolerance in Human Monocytes and Macrophages
Source: Front Immunol. 2020 Oct 5;11:550769. doi: 10.3389/fimmu.2020.550769 (PMC7573361; doi:10.3389/fimmu.2020.550769)

Supplementary Figure 1

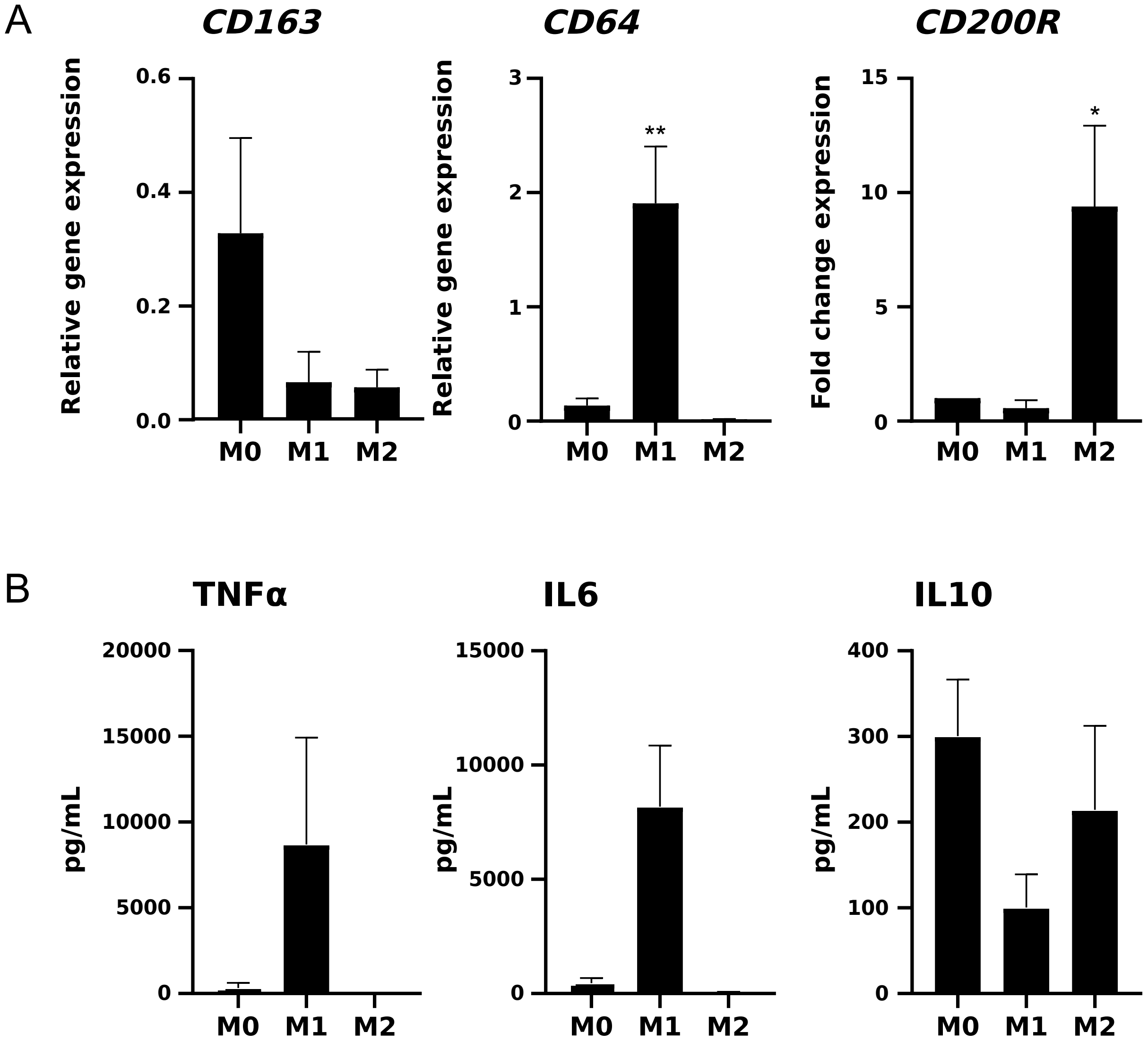

Supplementary Figure 2

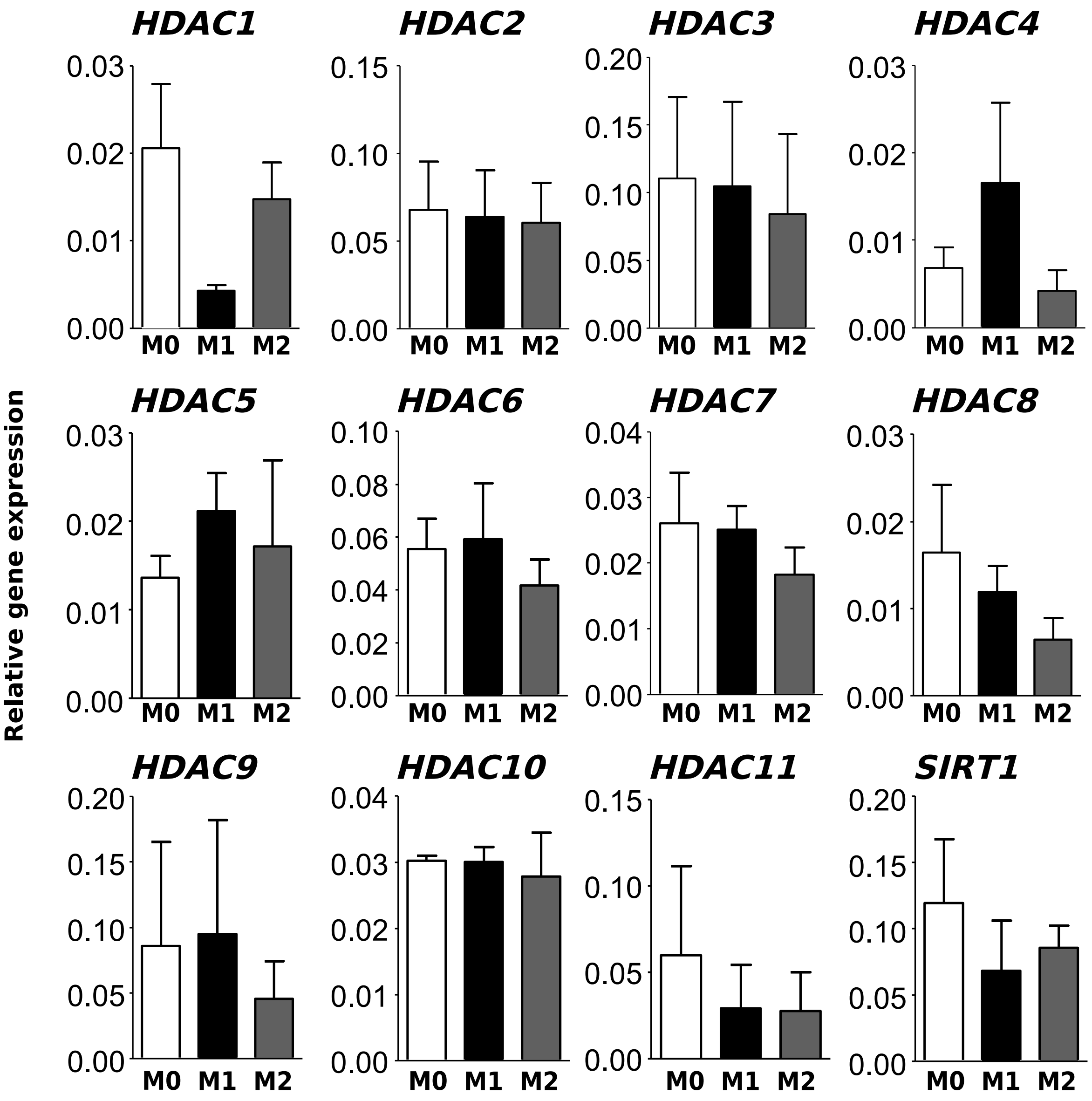

Supplementary Figure 3

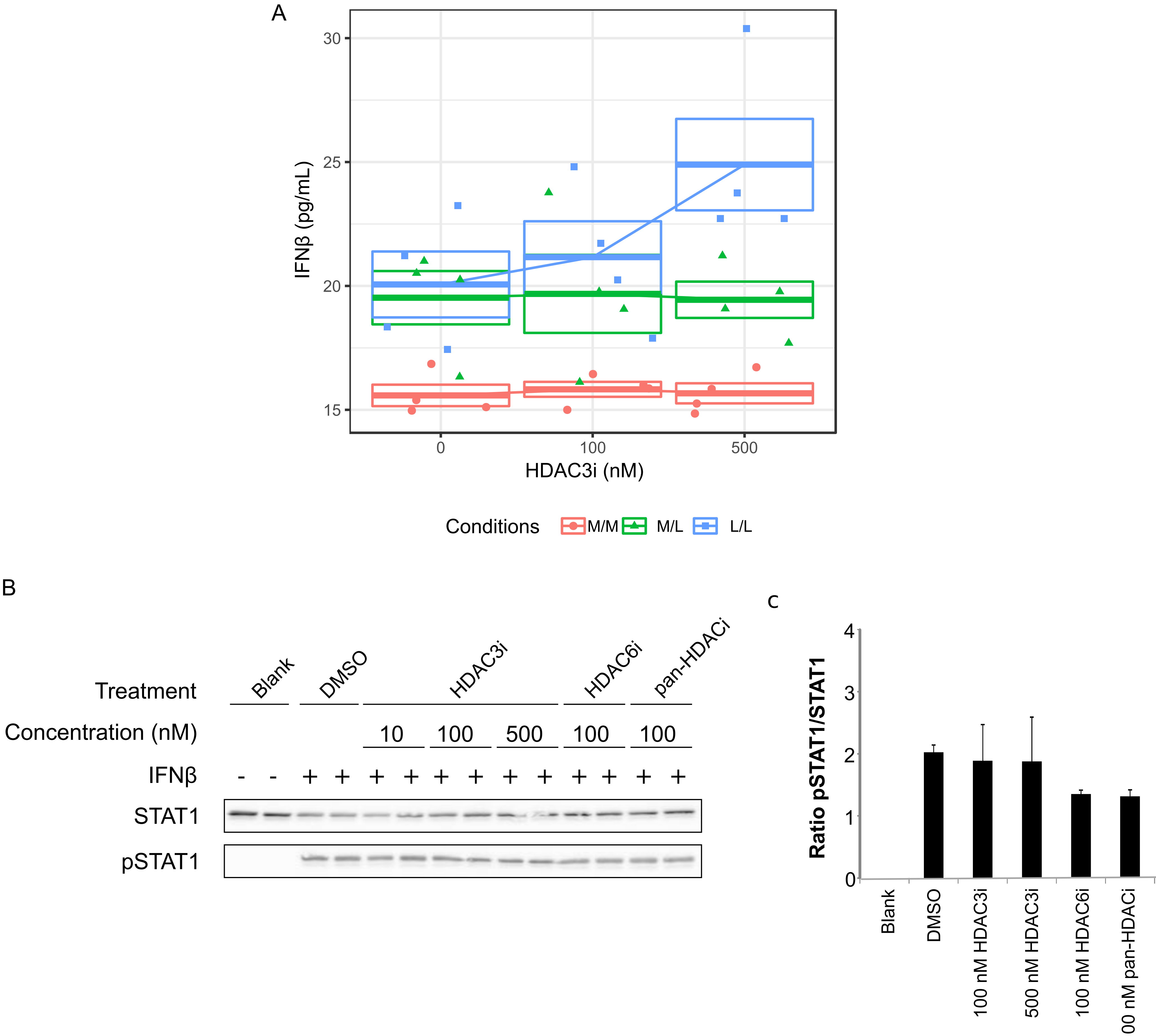

Supplementary figure 4

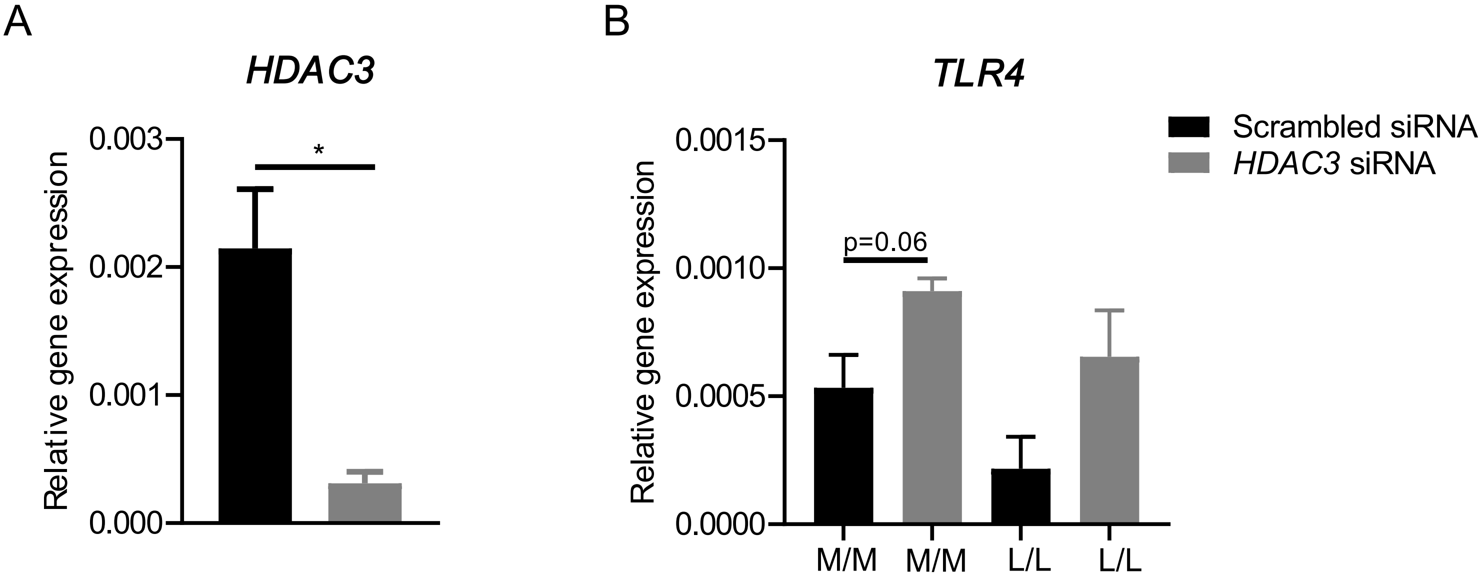

Supplementary figure 5

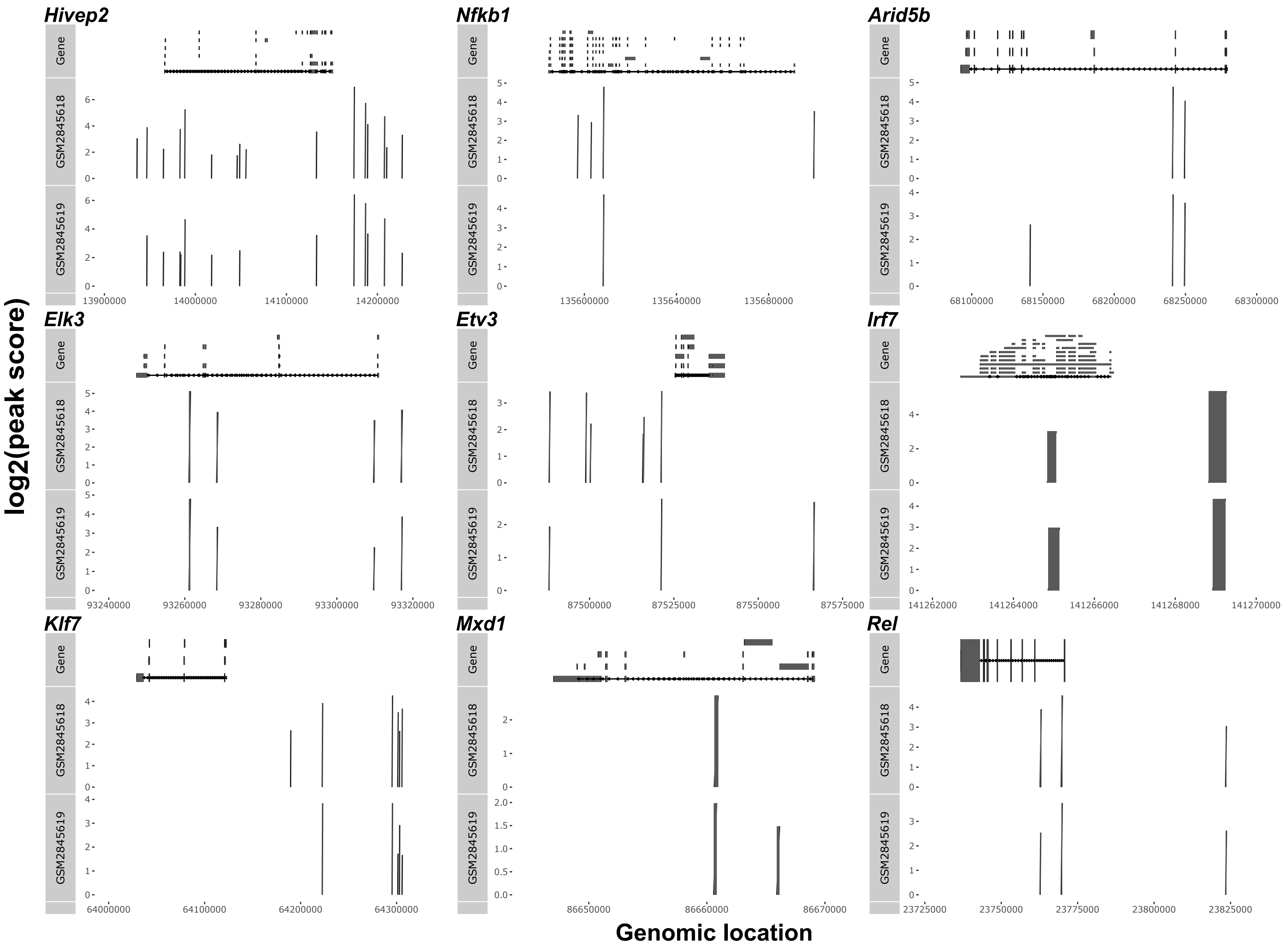

Supplementary figure 6

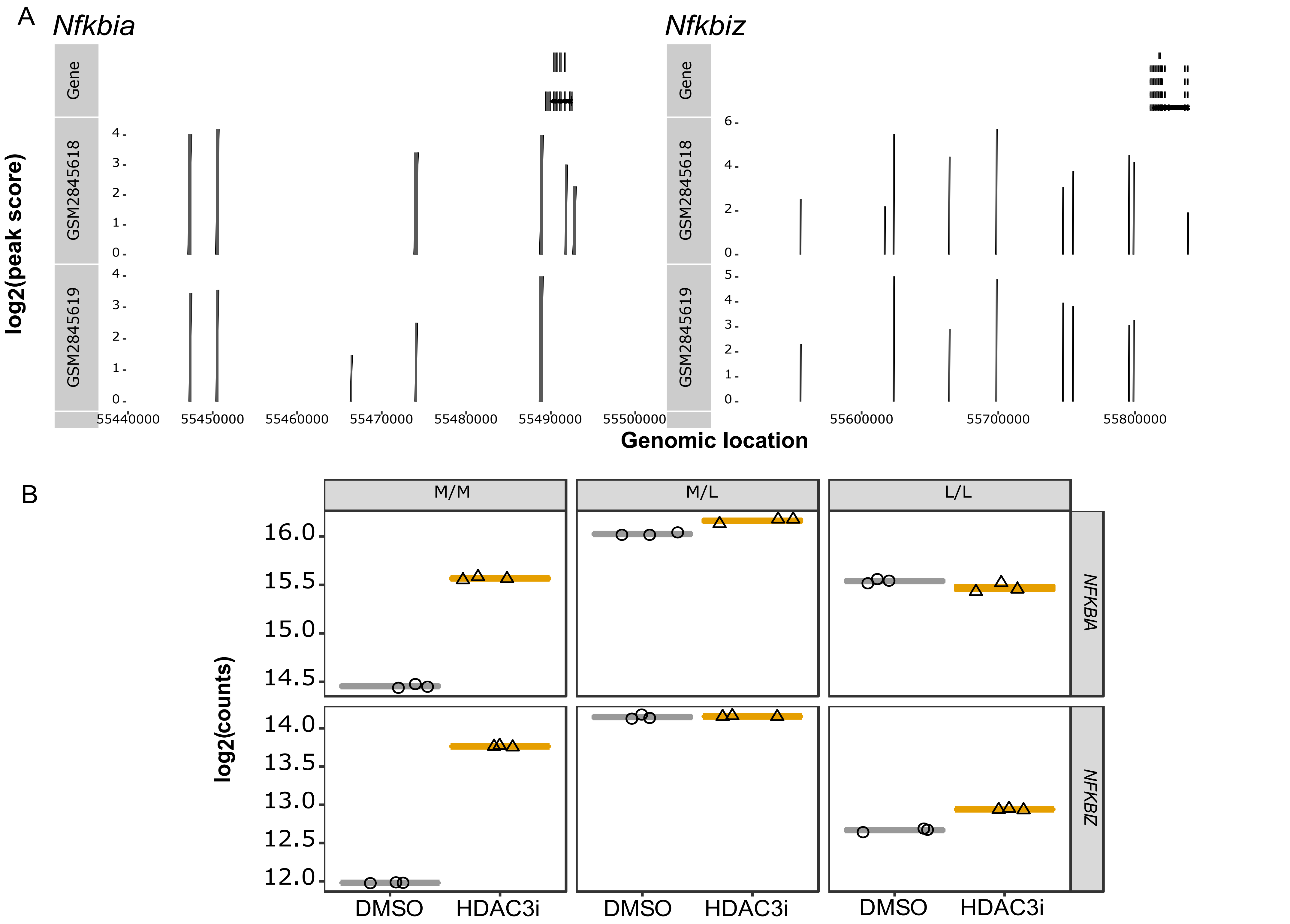

Supplement: Supplementary file 2 [file DataSheet_2.pdf]
