# Supplementary material for: HDAC3 Mediates the Inflammatory Response and LPS Tolerance in Human Monocytes and Macrophages
Source: Front Immunol. 2020 Oct 5;11:550769. doi: 10.3389/fimmu.2020.550769 (PMC7573361; doi:10.3389/fimmu.2020.550769)
Supplement: Supplementary file 3 [file Table_1.pdf]

Supplemental Table 1. Primer sequences. Primer sequences used in the quantitative PCR analysis of the genes of interest.

Gene	Forward (5'-3')	Reverse (5'-3')
GAPDH	GTCAGTGGTGGACCTGACCT	TGAGCTTGACAAAGTGGTCG
HPRT	AGTTCTGTGGCCATCTGCTT	GTTAAACAACAATCCGCCCA
36B4	TCATCAACGGGTACAAACGA	GCCTTGACCTTTTCAGCAAG
CD64	GCAGGAACACATCCTCTGAA	GTAACTGGAGGCCAAGCACT
CD200R	GAGCAATGGCACAGTGACTGTT	GTGGCAGGTCACGGTAGACA
CD163	ACATAGATCATGCATCTGTCATTTG	ATTCTCCTTGGAATCTCACTTCTA
HDAC1	CTTCCTGCTGAGTCCCTCAC	GGCACCCTTTATGGTTCAAA
HDAC2	AGGCCCCATAAAGCCACTGCC	AGCTCCAGCAACTGAACCGCC
HDAC3	CTGTGTAACGCGAGCAGAAC	GCAAGGCTTCACCAAGAGTC
HDAC4	GACGGTGCACTCGGAAGCCC	CTACCACGCAGCCCACAGCC
HDAC5	GTGACACCGTGTGGAATGAG	AGTCCACGATGAGGACCTTG
HDAC6	GGGTGCCAGCAGCCAGATCG	AGCAGGTGGGTGAGGTGGGC
HDAC7	GTCCTGGTGTCTGCTGGATT	AAGGGGATCCACCCTGTTAC
HDAC8	GCGTGATTTCCAGCACATAA	ATACTTGACCGGGGTCATCC
HDAC9	GCCCACAGGAACTTCTGACT	GAACTCTAAGCCAGATGGGG
HDAC10	TCCACCCGAGTACCTTTCAC	GATCCTGTGTAGCCCGTGTT
HDAC11	CGAGGCACCTAACATCCATT	TGCGCTACAAGAACTTTCCA
SIRT1	TCAGTGGCTGGAACAGTGAG	TCTGGCATGTCCCACTATCA

IL6	AGTGAGGAACAAGCCAGAGC	GTCAGGGGTGGTTATTGCAT
TLR4	CGGTGATAGCGAGCCACGCATT	ATATTAGGAACCACCTCCACGCAGGG
